# Six-Minute Walking Distance Improvement after Pulmonary Rehabilitation Is Associated with Baseline Lung Function in Complex COPD Patients: A Retrospective Study

**DOI:** 10.1155/2013/483162

**Published:** 2013-12-18

**Authors:** Andrea Zanini, Alfredo Chetta, Federico Gumiero, Sabrina Della Patrona, Silvia Casale, Elisabetta Zampogna, Marina Aiello, Antonio Spanevello

**Affiliations:** ^1^Division of Pneumology, IRCCS Rehabilitation Institute of Tradate, Salvatore Maugeri Foundation, via Roncaccio 16, 21049 Tradate, Italy; ^2^Department of Clinical and Experimental Medicine, University of Insubria, via Ravasi 2, 21100 Varese, Italy; ^3^Respiratory Disease and Lung Function Unit, Department of Clinical and Experimental Medicine, University of Parma, Padiglione Rasori, via Rasori 10, 43125 Parma, Italy

## Abstract

*Introduction*. Conflicting results have been so far reported about baseline lung function, as predicting factor of pulmonary rehabilitation (PR) efficacy. *Aim*. To ascertain whether or not baseline lung function could predict a benefit in terms of a significant change in 6-min walk test (6MWT) after PR. *Methods*. Seventy-five stable moderate-to-severe COPD inpatients with comorbidities (complex COPD), allocated to a three-week PR program, were retrospectively evaluated. Pulmonary function, 6MWT, dyspnea (BDI/TDI), and quality of life (EQ-VAS) were assessed before and after PR program. The patients were divided into two groups depending on the change in 6MWT (responders > 30 m and nonresponders ≤ 30 m). Logistic regression analysis was used. *Results*. After PR, 6MWT performance all outcome measures significantly improved (*P* < 0.01). Compared to nonresponders (*N* = 38), the responders (*N* = 37) had lower values in baseline lung function (*P* < 0.01). Logistic regression analysis showed that FEV_1_  <  50% pred and TL, CO <  50% pred were independent predictors of PR efficacy. *Conclusions*. Our study shows that in stable moderate-to-severe complex COPD inpatients, baseline lung function may predict the response to PR in terms of 6MWT. We also found that complex COPD patients with poor lung function get more benefit from PR.

## 1. Introduction

Pulmonary rehabilitation (PR) is widely used to treat COPD patients with different degrees of severity and prevalence of chronic comorbidities [1], bringing them benefits in terms of improved exercise capacity, symptoms, and quality of life, regardless of whether the setting is inpatient or ambulatory [2, 3]. Several studies have focused on identifying clinical and functional predictors of the beneficial effects of PR in COPD patients [4]. However, determining patients who may benefit from PR remains a debatable issue, with no conclusive data available.

To date, change in exercise performance is still considered one of the most important and easiest outcome measures adopted to evaluate the effects of PR in COPD patients [5]. Since PR is a comprehensive and multidimensional intervention, little is known as yet about the correlation existing between change in exercise capacity after PR and the predictive value of multiple factors potentially associated with this change [4]. Some of the baseline characteristics of COPD patients, such as arterial oxygenation [6], degree of dyspnea [7], body mass index (BMI) arterial partial pressure of oxygen (PaO_2_) [8], and health status [9], appear to be considered as predictors of PR, in terms of change in walking distance of 6-minute walking test (6MWT). In these studies [6–9], there has been the general observation that, in COPD patients, a poorer baseline condition, due to a higher magnitude of breathlessness, deconditioning in overweight, hypoxemia, or health status impairment, can leave greater possibility for improvement in exercise capacity after PR.

Up to now, conflicting results have been reported about the role of baseline lung function, as predicting factor of PR efficacy. According to some reports, the FEV_1_ baseline value appears to be irrelevant to predict benefits from PR in COPD patients [6, 7, 10, 11]. Other studies [8, 9] provided evidence that improvements in physiologic training response of PR program were positively associated with the degree of airflow limitation. By contrast, a recent study [12] showed that COPD patients with poor lung function get more benefit from PR in terms of endurance walking capacity.

The aim of this study was to examine in patients with clinically stable complex COPD the relationship between functional baseline parameters and exercise response to PR and to ascertain whether or not baseline lung function could predict a benefit in terms of a significant change in 6MWT after PR. We performed, therefore, a retrospective analysis from a database of inpatients with complex COPD who had undergone PR in a tertiary care center.

## 2. Methods

### 2.1. Design of the Study

A retrospective analysis was performed on data from COPD patients admitted to our rehabilitation center from January 1 to December 31, 2011.

Spirometry and blood gas analysis together with walking capacity, dyspnea, and HRQoL were measured in all patients at admission and at the end of the PR program.

Outcome measure was the change in walk distance after completing PR. Correlation between baseline variables and improvement in walk distance were also analyzed.

### 2.2. Subjects

We examined 151 COPD patients who attended an inpatient PR program. All patients were diagnosed with COPD according to the GOLD criteria [13]. Patients suffering from acute exacerbation over the previous four weeks were excluded, as well as patients who were not able to perform a 6MWT. Patients who did not complete the PR program, for COPD exacerbation, or any unstable medical condition, were also excluded. Contraindications for participation in the PR program included musculoskeletal disorders, malignant diseases, unstable cardiac condition, and lack of compliance to the program. Finally, 75 patients were considered for the study.

Individuals' self-reported comorbidities, as assessed by the Charlson Index [14] which assigns to each disease a score that is proportional to the disease related risk of death, were retrieved by the medical files. The Charlson Index was computed during the hospital stay by the physician in charge of each admitted patient.

All of patients were exsmokers and were receiving regular pharmacologic treatment (inhaled long-acting *β*2-agonists in 62 patients, tiotropium in 70 patients, and inhaled corticosteroids in 44 patients). Eleven patients were under long-term oxygen therapy and 13 out of 75 had two or more exacerbations in the preceding year and were classified as frequent exacerbators.

In all patients, the clinical and functional assessment had been undertaken for clinical reasons at the request of the patient's clinician and approval to report these data has been given by our ethical review board. All participants' data were analyzed and reported anonymously. No extramural funding was used to support this study.

### 2.3. Pulmonary Function Tests and Arterial Blood Gas Analysis

VC, FEV_1_, TLC, and RV were measured by means of a flow-sensing spirometer and a body plethysmograph connected to a computer for data analysis (Masterlab, Jaeger, Wurzburg, Germany). TL, CO was measured by the single breath method using a mixture of carbon monoxide and methane (Sensor Medics, Yorba Linda, USA). VC, FEV_1_, TLC, RV, and TL, CO were expressed as a percentage of the predicted values, which were obtained from regression equations by Quanjer et al. [15] and Cotes et al. [16]. FEV_1_/VC and RV/TLC ratios were taken as indices of airway obstruction and lung hyperinflation, respectively. 

PaO_2_ and PaCO_2_ were measured immediately after sampling from a puncture of the radial artery at rest (Gas analyzer ABL 330; Radiometer, Copenhagen, Denmark).

### 2.4. Walking Capacity

Walking capacity was evaluated by means of the distance covered during a 6MWT according to the ATS statement [17]. In all patients, the change in distance covered during 6MWT (Δ6MWD) after PR was recorded. Before and immediately after the 6MWT, patients rated the magnitude of their perceived breathlessness and of their leg fatigue using a 1–10-point Borg scale.

### 2.5. Dyspnea and Health Status-HRQoL

Dyspnea was assessed by the baseline/transitional dyspnea index (BDI/TDI) [18]. Health status-HRQoL of patients was evaluated by the VAS component of EQ-5D, reflecting their perceived health state, where 0 meant the “worst imaginable health state” and 100 meant the “best imaginable health state” [19].

### 2.6. Pulmonary Rehabilitation Program

Patients underwent a comprehensive PR program consisting of (a) exercise training, (b) verbal inputs stressing the need for adherence to therapy, (c) educational support, and (d) a nutritional and psychological counseling, if needed. According to the guidelines recommendations, the PR program was completely tailored to suit the needs of the individual [2, 3]. The program consisted of 12 sessions completed over a 3-week period, including (a) aerobic exercise training (cycling, walking, and/or arm exercise), (b) respiratory muscle training, (c) breathing exercise, (d) postural exercises, and (e) upper- and lower-body muscle strength training exercise. Exercises were graded, being their intensity weekly increased as the patient progressed in the PR [20]. The exercise program was supervised by a chest physiotherapist. Patients with chronic respiratory failure were provided with oxygen during the exercise sessions.

### 2.7. Statistical Analysis

Data are reported as mean ± standard deviation (SD), unless otherwise specified. The distribution of variables was assessed by means of Kolmogorov-Smirnov Goodness-of-Fit test. Relationships between variables were assessed by Pearson's correlation coefficient (*r*) and linear regression analysis. Comparisons between variables were determined by unpaired *t*-test and *χ* ^2^ test, when appropriate.

In order to evaluate the role of baseline lung function parameters to predict the PR benefit, the COPD patients were subdivided into different subgroups according to FEV_1_ (≥50% pred, *N* = 44 and <50% pred, *N* = 31), RV (≥160% pred, *N* = 36 and <160% pred, *N* = 39), TL, CO (≥50% pred, *N* = 53 and <50% pred, *N* = 22), and PO2 (≥70 mm Hg, *N* = 39 and <70 mm Hg, *N* = 36). The efficacy of PR was expressed by the significant Δ6MWD, consisting in an increase in walked distance greater than 30 meters after PR, which is considered as a minimal clinically important difference (MCID) [21]. According to whether or not the patients reached a MCID, they were classified as responders (Δ6MWD > 30 m, *N* = 37) and nonresponders (Δ6MWD ≤ 30 m, *N* = 38). Logistic regression analysis was then performed to test the association between the baseline lung function parameters, as binary independent variables, and the significant Δ6MWD, as a binary dependent variable. Odds ratios are presented with 95% confidence intervals. 

A *P* value < 0.05 was considered as significant.

## 3. Results

Characteristics of COPD patients are reported in Table 1. According to GOLD criteria 9, 35, 27, and 4 out of 75 patients had mild, moderate, severe, and extremely severe airflow obstruction, respectively. After PR, a significant improvement in 6MWT, TDI, and EQ-VAS was found in all patients. The 6MWT improved by 35 ± 39 meters (from 440 ± 102 to 475 ± 91, *P* < 0.001). Dyspnea showed a clinically significant reduction (from BDI 7.1 ± 2.3 to TDI 3.8 ± 2.1), corresponding to a change of ≥1 unit in 94% of the patients. EQ-VAS improved by 15.3 ± 12 (from 57.8 ± 18 to 72.7 ± 15.2, *P* < 0.001).

After PR, there was a very modest, though statistically significant, increase in both FEV_1_ and VC (*P* < 0.001 and *P* = 0.006, resp.) in responders and in FEV_1_ in nonresponders (*P* = 0.02).

As compared to nonresponders, responders were significantly younger with a worse respiratory function and showed a higher percentage of frequent exacerbators (27% versus 8%, *P* = 0.031). At baseline, responders were more dyspnoeic than nonresponders (BDI, 6.4 ± 2.2 versus 7.7 ± 2.3, *P* = 0.021) and at baseline experienced a higher dyspnea (Borg scale, 4.6 ± 2.3 versus 3.1 ± 1.6, *P* = 0.004) and a higher leg fatigue (Borg scale 4.1 ± 2.4 versus 3.1 ± 1.5, *P* = 0.036) during the 6MWT than nonresponders. No differences were observed in EQ-VAS between the two groups at baseline. Fifty-three out of 75 patients participated at a previous PR (71%). They did not differ in walking distance, as compared to the remaining patients.

**Figure 1:**
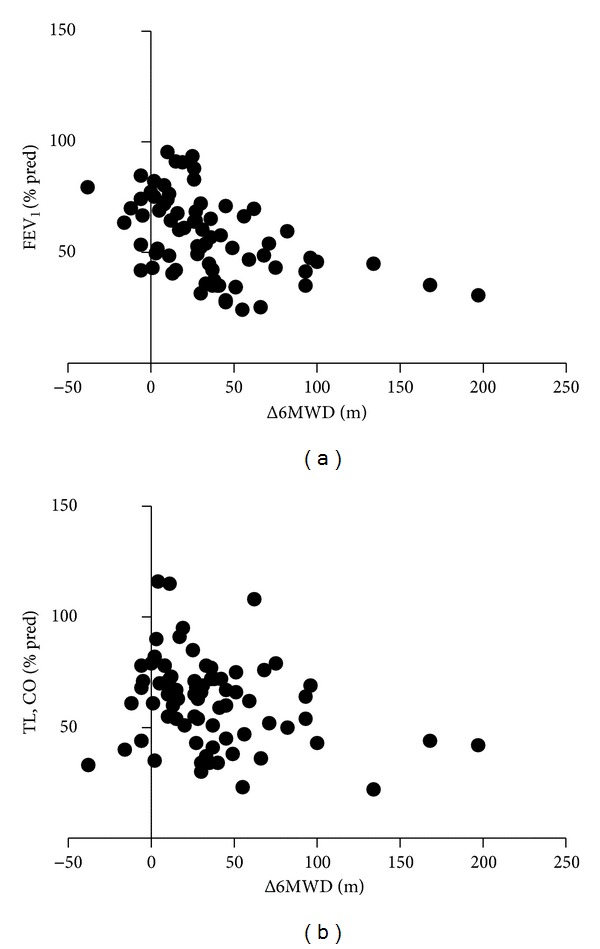
Relationship between the change in six-minute walking distance after pulmonary rehabilitation and FEV_1_ (a) and TL, CO (b).

In all patients, Δ6MWD was inversely related to baseline values of FEV_1_% predicted (*r* = −0.50) (Figure 1), VC% predicted (*r* = −0.45), IC (*r* = −0.38), FEV_1_/VC (*r* = −0.33), PaO_2_ (*r* = −0.30), and TL, CO% predicted (*r* = −0.25) (Figure 1) and directly related to baseline values of RV/TLC (*r* = 0.40), RV% predicted (*r* = 0.33). Moreover, in all patients and in responders group Δ6MWD was inversely related to baseline values of 6MWD (*r* = −0.47 and *r* = −0.46, resp.).

Logistic regression analysis showed that, in all patients, the significant change in 6MWT was significantly associated with FEV_1_ and TL, CO values but not with RV and PaO_2_ values (Table 2).

## 4. Discussion

In this retrospective study, we examined the role of lung function and clinical parameters at baseline in determining benefits after a 3-week PR program in 75 moderate-to-severe and stable complex COPD patients. As expected, we found an improvement in PR outcomes in all patients. In addition, we observed that a worse baseline lung function was associated with a better response in walking capacity to PR. Notably, airflow obstruction and lung diffusion were independent predictors of benefit, in terms of the fact that patients with FEV_1_ and TL, CO less than 50% of predicted values reached the MCID of 6MWT after PR, as compared to the other subgroups of patients.

The relationship between change in exercise capacity after PR and baseline clinical and functional characteristics of COPD patients has been extensively investigated [6–12]; however, the results are discordant and difficult to interpret. The finding that emerges most frequently from these studies [6, 7, 10, 11] is the negligible value of the baseline lung function in determining benefits after PR. Furthermore, when a relationship between lung function and change in exercise capacity was observed, patients with less severe obstruction showed greater improvements in exercise tolerance [8, 9].

In COPD patients recovering from an acute exacerbation, Cilione et al. retrospectively studied the predictors of change in exercise capacity after comprehensive COPD inpatient rehabilitation [6]. They found that baseline values of 6MWD and arterial oxygenation had the most consistent correlation with change in 6MWD [6]. Garrod et al. [7] studied the predictors of success and failure in pulmonary rehabilitation in a heterogeneous group of COPD patients. They recruited their patients from primary and secondary care, who followed either out-patient or home-based PR with relation to the severity degree of the disease and did not find any relationship between baseline lung function and change in 6MWD [7]. Interestingly, they observed that patients with lower FEV_1_ showed greater improvement in quadriceps strength [7]. Vagaggini et al. [8] retrospectively evaluated clinical predictors of the efficacy of a pulmonary rehabilitation program in moderate-to-severe outpatients with complex COPD and by logistic regression analysis only BMI and PaO_2_ were positively associated with the improvement in 6MWD [8]. More recently, in a large cohort of moderate-to-severe COPD patients, van Ranst et al. found a weak positive correlation between baseline values of FEV_1_ and FEV_1_/VC and the improvement in 6MWD [9]. In this study, FEV_1_/VC, baseline WD, and peak oxygen uptake at incremental cycle exercise test contributed to explain a modest 19% of the variance of change in 6MWD [9].

In contrast to these data, we found that our complex COPD patients with worse baseline lung function, both in terms of airflow obstruction and in terms of diffusion lung capacity, gained greater improvement in walking capacity. Differences in participants and in PR setting might explain this discrepancy. Our patients were followed in an inpatient PR program in a specialized rehabilitation center. Thus, our patient sample comprised a considerable portion of severe to extremely severe patients (41%), including even oxygen-dependent patients. It is conceivable that patients with poor baseline lung function are at risk to enter a downward spiral of dyspnea, sedentariness, demotivation, and finally deconditioning [22]. On the other hand, these patients may show a larger capacity of improvement after PR, as compared to patients with more preserved lung function and exercise capacity. It is of note that our responder patients had a shorter 6MWD and a greater exertion dyspnea and leg fatigue than nonresponder patients; in addition, the percentage of patients with two or more exacerbations was significantly higher in the former group of patients than in the latter one.

Interestingly, we observed a significant but very modest clinical impact on dynamic lung volumes in both groups, with a higher magnitude in responders group. Even though the exercise program included a nutritional training as a component, it is difficult to attribute the improvements in ventilator capacity to this aspect per se. A more likely explanation relates to indirectly derived educational benefits in the use of inhaled medications.

Our results are in line with the other studies [12, 23]. Plankeel et al. [23] analyzed the change in exercise capacity after PR in a large population of nonhypoxemic patients with COPD. The patients were classified into subgroups based on the primary limitation seen on exercise testing, such as ventilator limited, cardiovascular limited, mixed ventilatory/cardiovascular limited, and noncardiopulmonary-limited. Interestingly, the authors found that the ventilatory limited group had a marked improvement in walk distance after PR and the degree of improvement was similar to the groups without ventilatory limitation [23]. Recently, by means of the cluster analysis Altenburg et al. [12] have investigated whether or not there is a patient profile among the COPD population, associated with the improvement in endurance walking capacity after PR. They identified a cluster profile of patients characterized by a larger improvement in walking capacity, assessed by endurance shuttle walk test, which was associated with poor baseline lung function consisting in high TLC and RV/TLC and low FEV_1_ values [12].

Finally, it is of note that individual comorbidities of our patients did not preclude effectiveness of PR course. These findings confirm the feasibility of our programme, which reproduces the internationally shared standards, and are in line with the results of Crisafulli and colleagues [24], which observed that, among all the individual comorbidities, either alone or in combination, only the presence of osteoporosis was independently associated with poorer rehabilitation outcomes.

In conclusion, our study shows that complex COPD patients with worse lung function, that is, with FEV_1_ and TL, CO values less than 50% of predicted, seem to benefit more from pulmonary rehabilitation, in particular reference to change in six-minute walk distance. This finding suggests that complex COPD patients with more severe pulmonary impairment not only should not be excluded from rehabilitation programs but also may have the best results.

## Figures and Tables

**Table 1:** Subjects' characteristics.

	All patients (*n* = 75)	Responders (*n* = 37)	Nonresponders (*n* = 38)	*P* value*
Age (yrs)	71 ± 8	69 ± 8	73 ± 7	0.020
Gender (F, M)	11, 64	6, 31	5, 33	0.708
FEV_1_ (% pred)	57 ± 18	45 ± 14	68 ± 15	<0.001
VC (% pred)	85 ± 16	78 ± 14	92 ± 15	<0.001
FEV_1_/VC (%)	50 ± 12	45 ± 11	55 ± 10	<0.001
RV (% pred)	162 ± 43	178 ± 43	147 ± 37	0.002
TLC (% pred)	113 ± 17	115 ± 18	111 ± 16	0.256
RV/TLC (%)	57 ± 9	61 ± 9	54 ± 7	<0.001
IC (liters)	2.2 ± 0.6	2 ± 0.6	2.4 ± 0.5	<0.001
TL, CO (% pred)	61 ± 20	55 ± 19	67 ± 19	0.007
BMI (Kg/m^2^)	29.7 ± 5.3	29 ± 6	30 ± 5	0.419
PaO_2_ (mm Hg)	71.2 ± 8.2	69 ± 5	73 ± 10	0.026
PaCO_2_ (mm Hg)	38.5 ± 4.5	39 ± 5	38 ± 4	0.070
Charlson Index	1.6 ± 0.9	1.8 ± 1	1.4 ± 0.8	0.084

*Responders versus nonresponders.

**Table 2:** Odds ratios (95% confidence intervals) by regression logistic analysis of FEV_1_ < 50% pred, TL, CO < 50% pred, RV > 160% pred, and PaO_2_ < 70 mm Hg for COPD responder to PR.

	OR (95% CI)	*P* value
FEV_1_ < 50% pred	5.740 (1.782–18.491)	0.003
TL, CO < 50% pred	4.001 (1.151–13.906)	0.028
RV > 160% pred	2.255 (0.717–7.090)	0.164
PaO_2_ < 70 mm Hg	1.147 (0.383–3.439)	0.806

## Abbreviations

6MWT: Six-minute walking test
6MWD: Six-minute walking distance
Δ6MWD: Six-minute walking distance change
PR: Pulmonary rehabilitation
BMI: Body mass index
FEV_1_: Forced respiratory volume in one second
VC: Vital capacity
TLC: Total lung capacity
RV: Residual volume
TL, CO: Transfer factor of the lung for carbon monoxide
PaO_2_: Arterial partial pressure of oxygen
PaCO_2_: Arterial partial pressure of carbon dioxide
HRQoL: Health-related quality of life
BDI/TDI: Baseline dyspnea index/transitional dyspnea index
EQ-VAS: EuroQol-visual analogue scale
MCID: Minimal clinically important difference.
